# Supplementary figures and images for: Systematic analysis of membrane contact sites in Saccharomyces cerevisiae uncovers modulators of cellular lipid distribution
Source: eLife. 2022 Nov 10;11:e74602. doi: 10.7554/eLife.74602 (PMC9648973; doi:10.7554/eLife.74602)

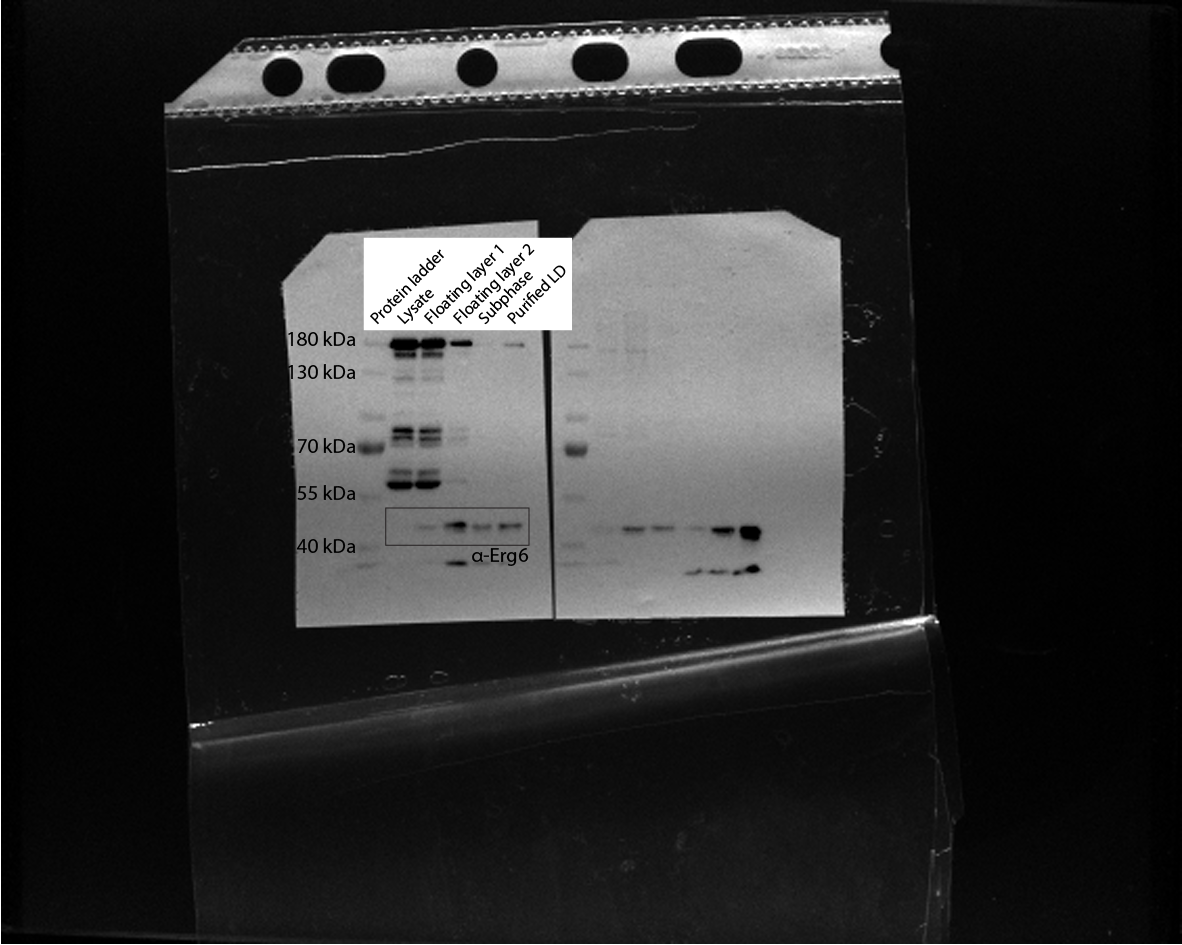

Supplement: Figure 3—source data 4. [file elife-74602-fig3-data4.zip › Figure 3-source data 4/Figure 3F anti-Erg6_blot-labelled blot.tif]

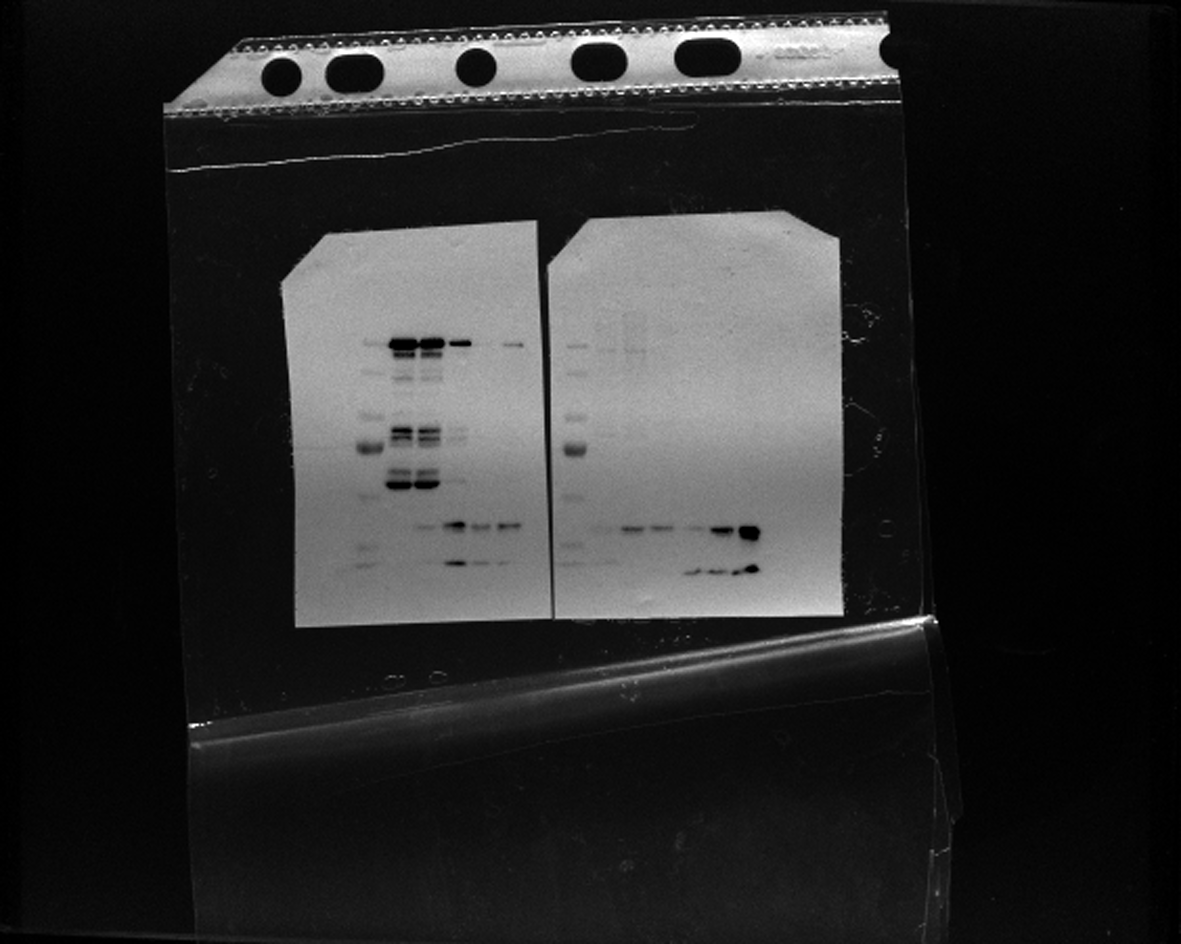

Supplement: Figure 3—source data 4. [file elife-74602-fig3-data4.zip › Figure 3-source data 4/Figure 3F anti-Erg6_blot-raw blot.tif]

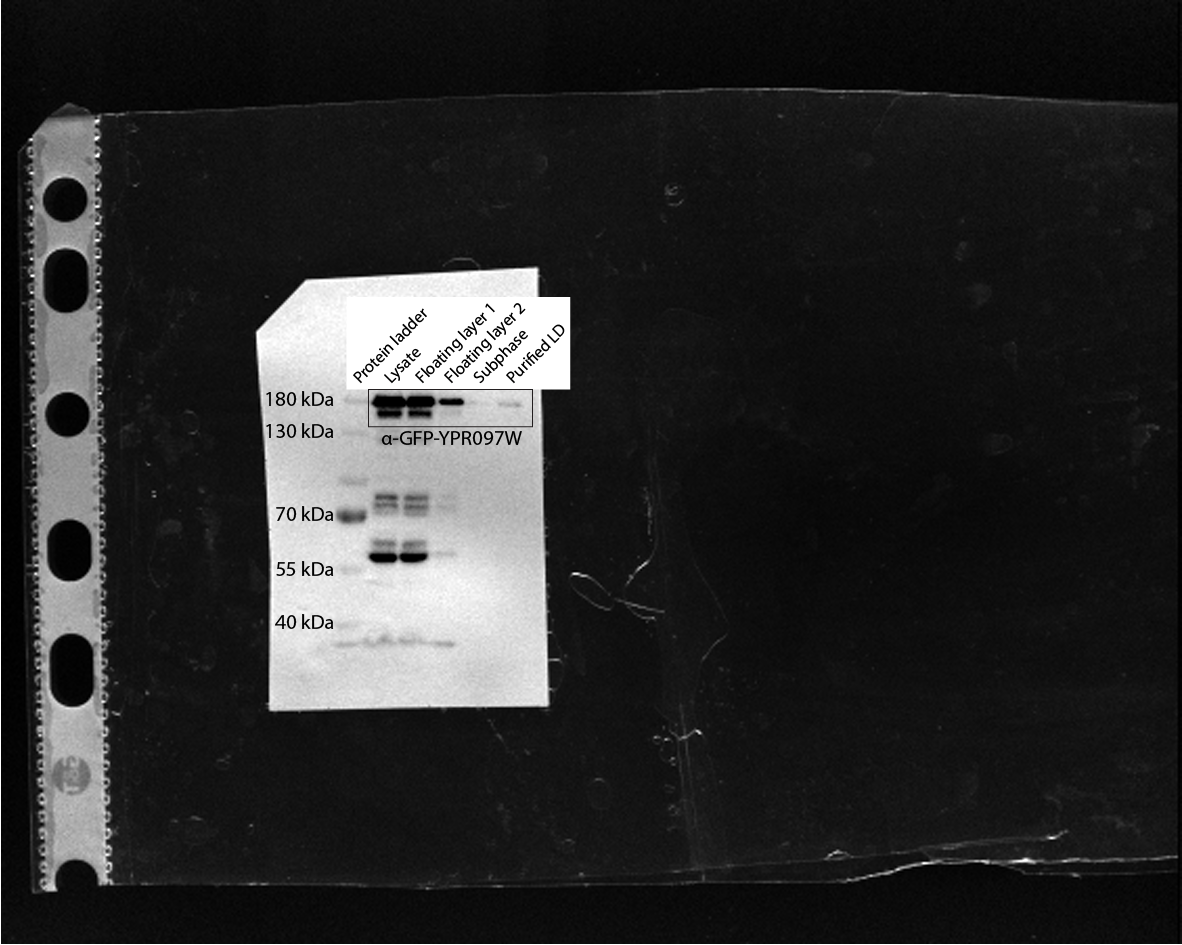

Supplement: Figure 3—source data 4. [file elife-74602-fig3-data4.zip › Figure 3-source data 4/Figure 3F anti-GFP-YPR097W-labelled blot.tif]

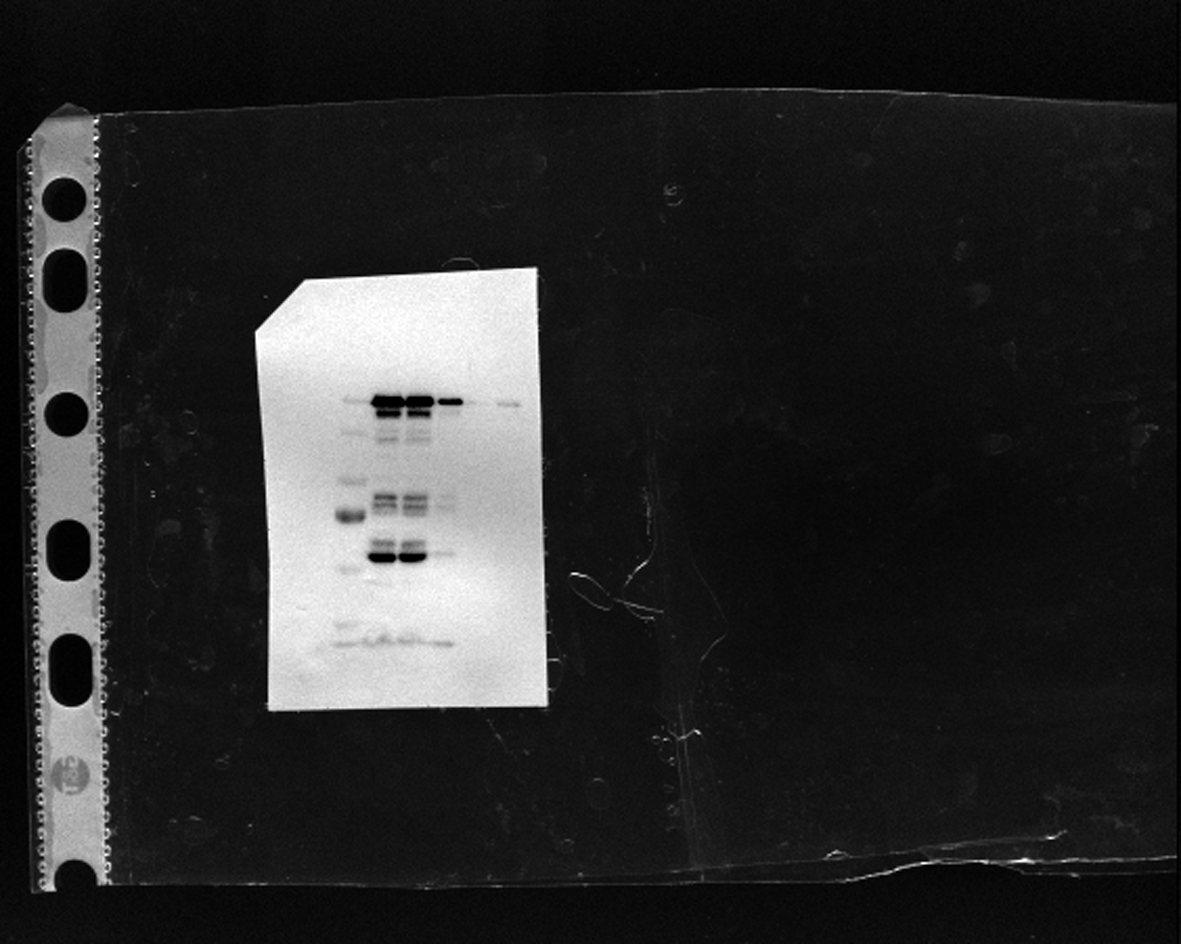

Supplement: Figure 3—source data 4. [file elife-74602-fig3-data4.zip › Figure 3-source data 4/Figure 3F anti-GFP-YPR097W-raw blot.tif]

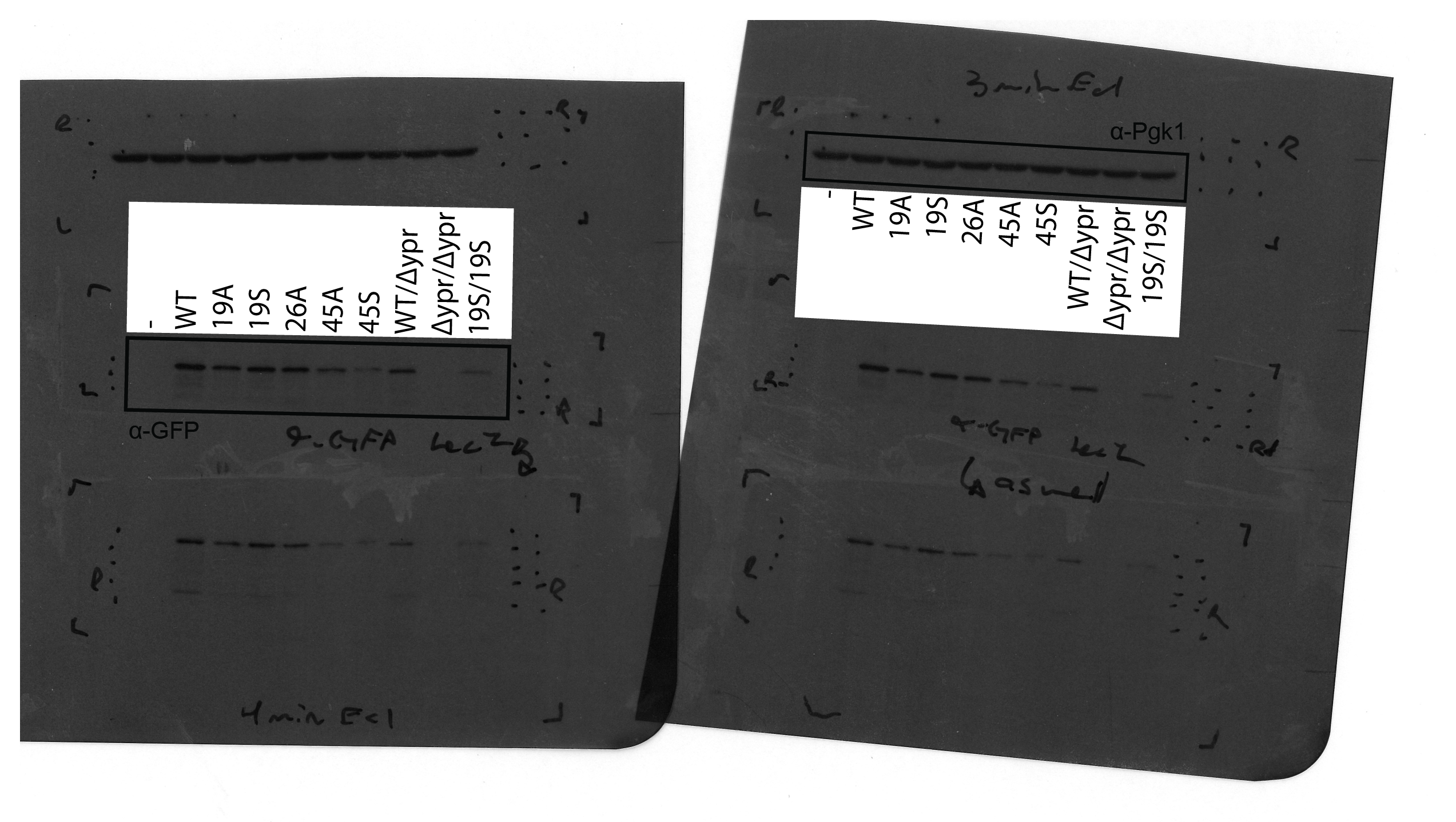

Supplement: Figure 4—figure supplement 2—source data 1. [file elife-74602-fig4-figsupp2-data1.zip › Figure 4-figure supplemente 2-source data 1/Figure 4-figure supplement 2-labelled blot.tif]

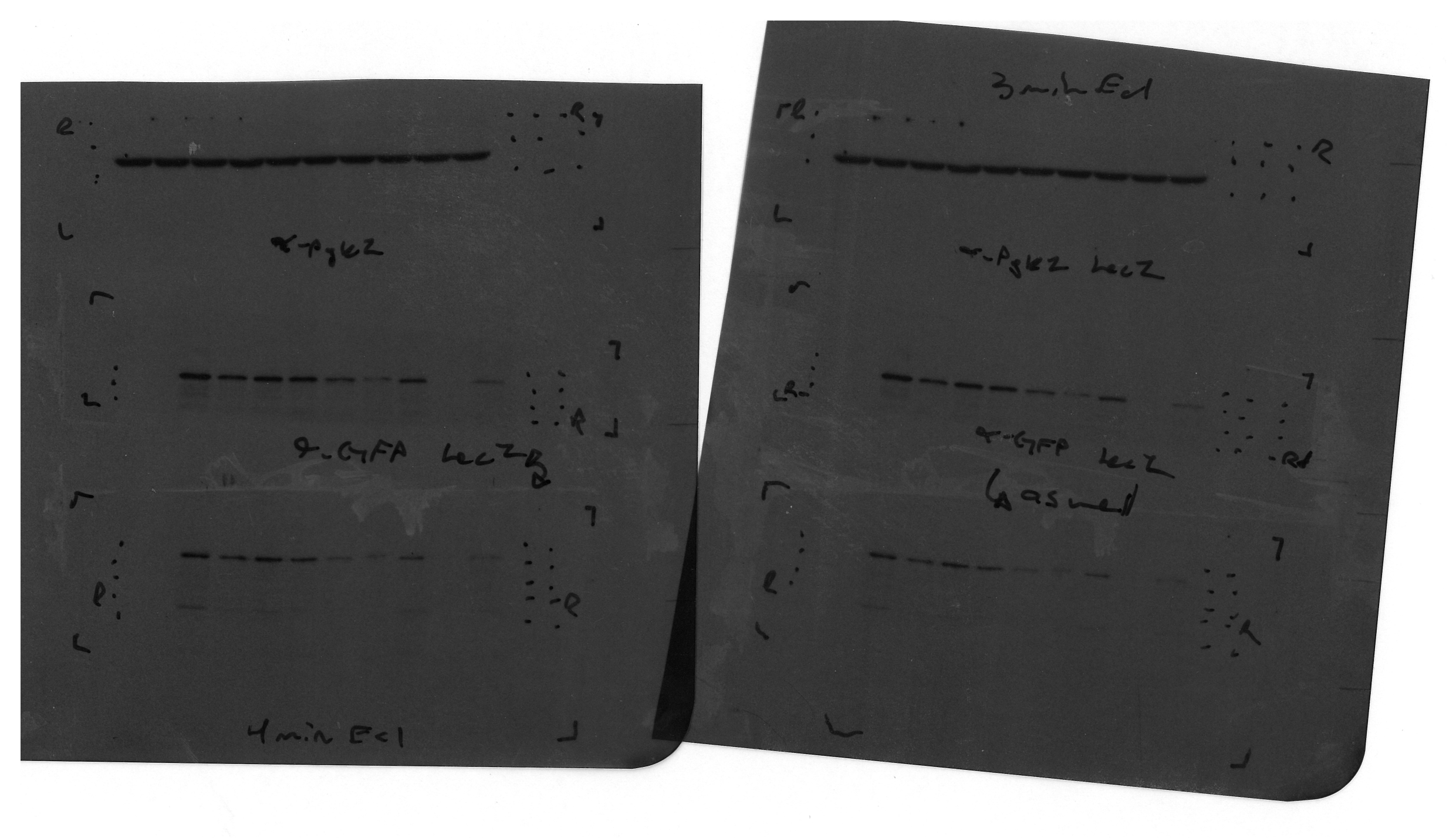

Supplement: Figure 4—figure supplement 2—source data 1. [file elife-74602-fig4-figsupp2-data1.zip › Figure 4-figure supplemente 2-source data 1/Figure 4-figure supplement 2-raw blot.tif]

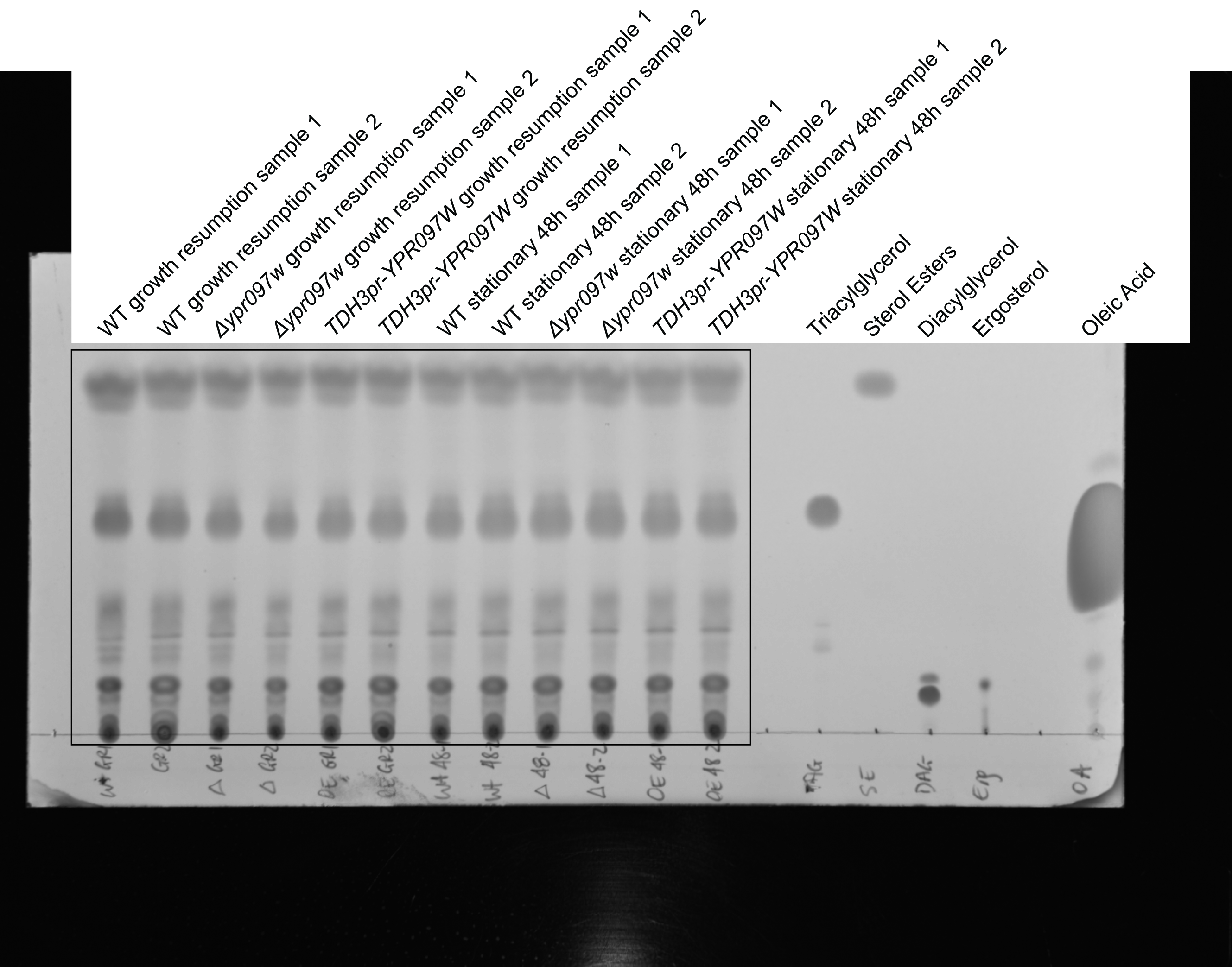

Supplement: Figure 5—figure supplement 1—source data 2. [file elife-74602-fig5-figsupp1-data2.zip › Figure 5-figure supplemente 1-source data 2/Figure 5-figure supplement 1-GR_Stationary 48h-labelled blot.tif]

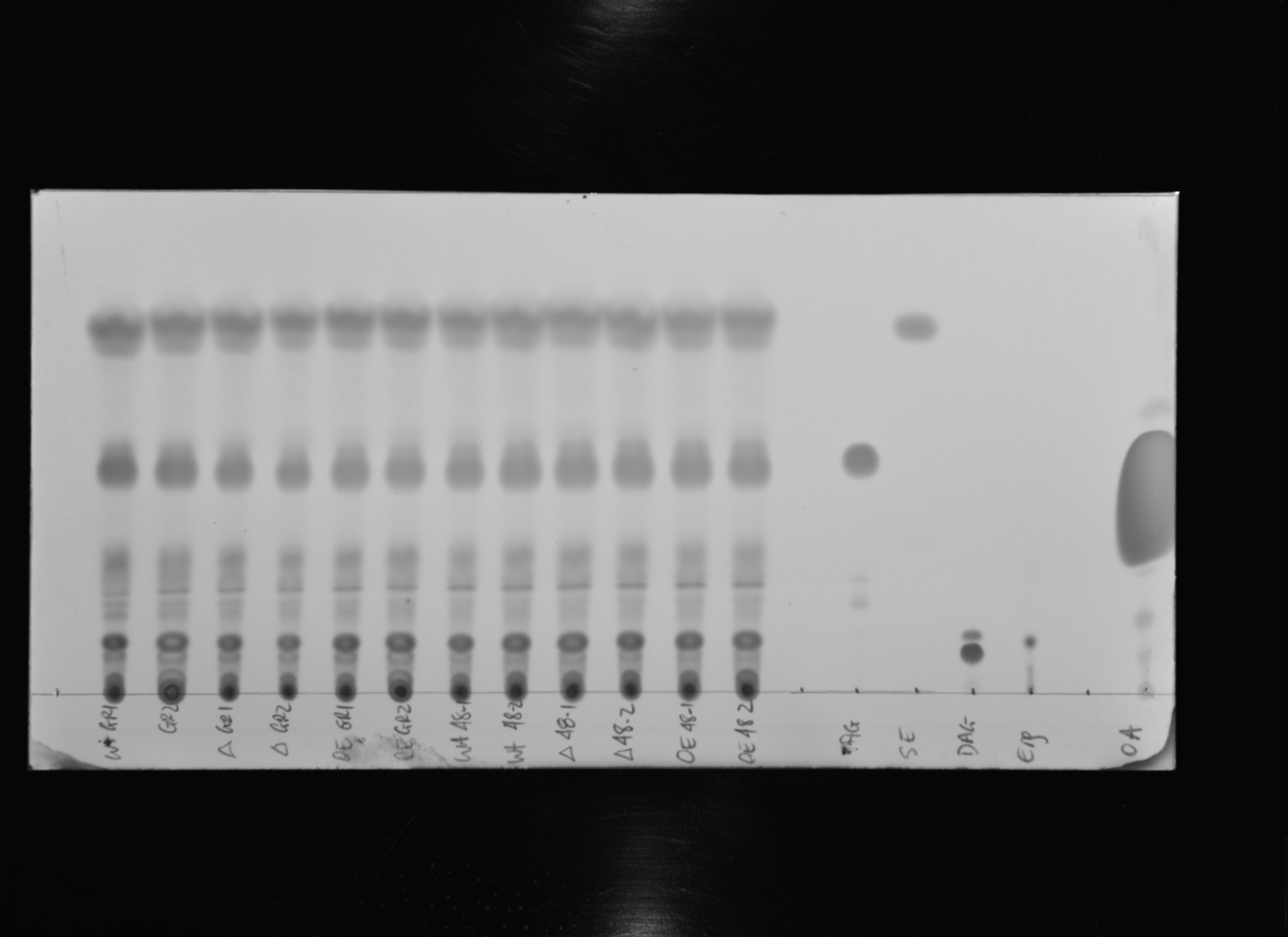

Supplement: Figure 5—figure supplement 1—source data 2. [file elife-74602-fig5-figsupp1-data2.zip › Figure 5-figure supplemente 1-source data 2/Figure 5-figure supplement 1-GR_Stationary 48h-raw blot.tif]

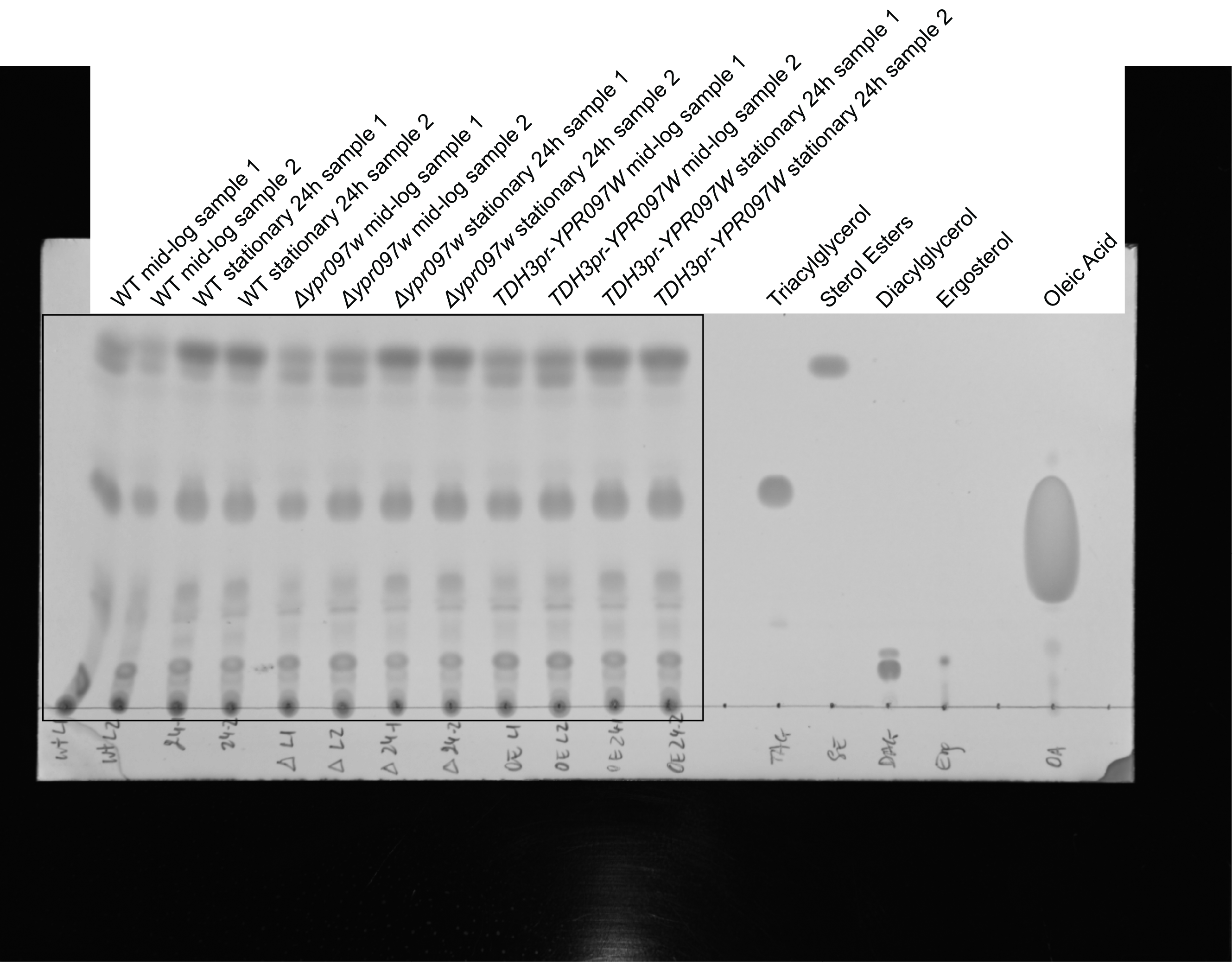

Supplement: Figure 5—figure supplement 1—source data 2. [file elife-74602-fig5-figsupp1-data2.zip › Figure 5-figure supplemente 1-source data 2/Figure 5-figure supplement 1-Mid-log_Stationary 24h-labelled blot.tif]

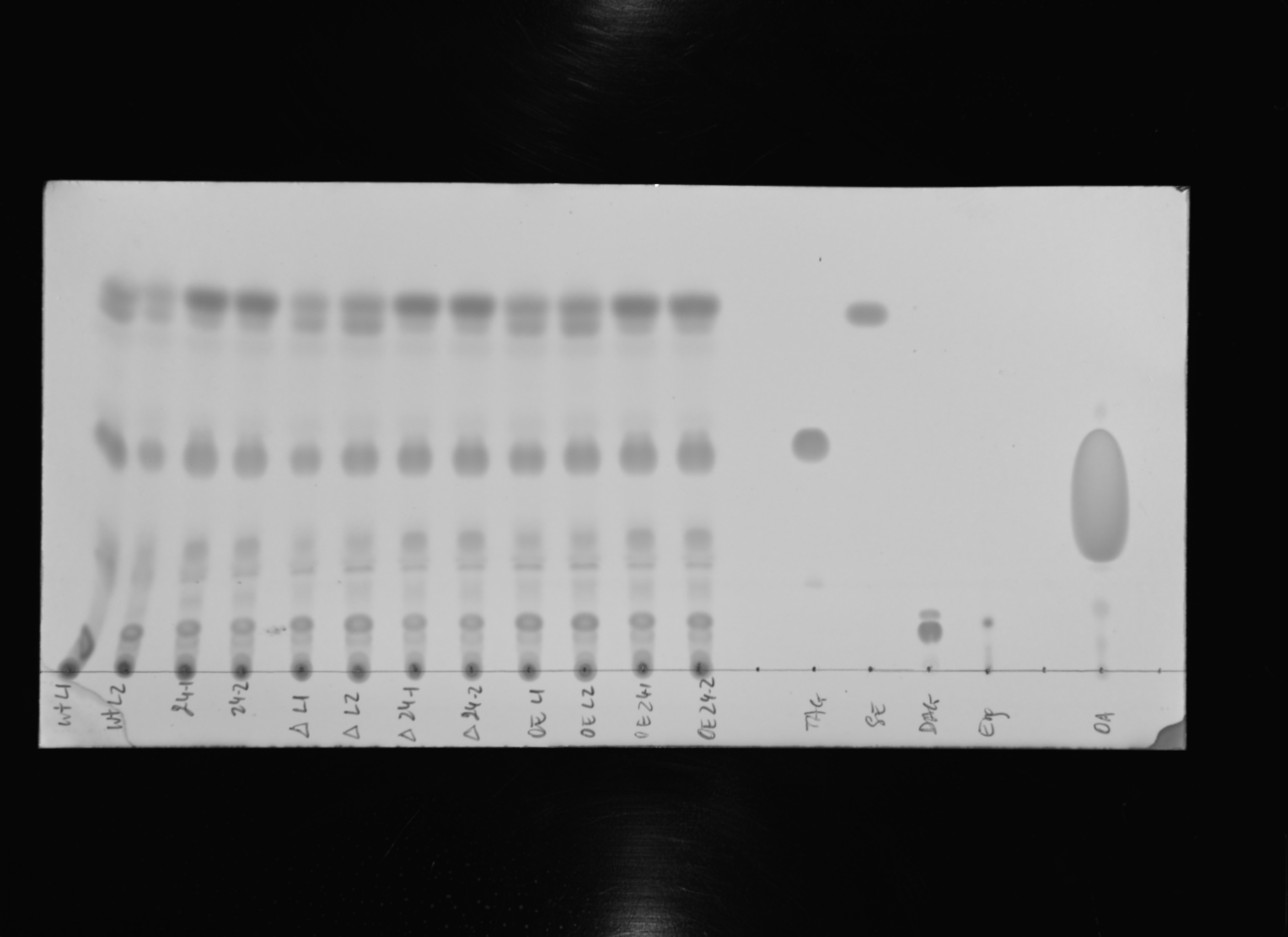

Supplement: Figure 5—figure supplement 1—source data 2. [file elife-74602-fig5-figsupp1-data2.zip › Figure 5-figure supplemente 1-source data 2/Figure 5-figure supplement 1-Mid-log_Stationary 24h-raw blot.tif]
